# Pyruvate Supports RET-Dependent Mitochondrial ROS Production to Control *Mycobacterium avium* Infection in Human Primary Macrophages

**DOI:** 10.3389/fimmu.2022.891475

**Published:** 2022-07-06

**Authors:** Lisa Marie Røst, Claire Louet, Per Bruheim, Trude Helen Flo, Alexandre Gidon

**Affiliations:** ^1^ Department of Biotechnology and Food Science, Faculty of Natural Sciences, Norwegian University of Science and Technology (NTNU), Trondheim, Norway; ^2^ Center of Molecular Inflammation Research (CEMIR), Faculty of Medicine and Health Sciences, Norwegian University of Science and Technology (NTNU), Trondheim, Norway; ^3^ Department of Clinical and Molecular Medicine, Faculty of Medicine and Health Sciences, Norwegian University of Science and Technology (NTNU), Trondheim, Norway

**Keywords:** *Mycobacterium avium* infection, innate immunity, human primary macrophages, glycolysis, pyruvate, reverse electron transport, mitochondrial ROS, mitochondrial pyruvate

## Abstract

Macrophages deploy a variety of antimicrobial programs to contain mycobacterial infection. Upon activation, they undergo extensive metabolic reprogramming to meet an increase in energy demand, but also to support immune effector functions such as secretion of cytokines and antimicrobial activities. Here, we report that mitochondrial import of pyruvate is linked to production of mitochondrial ROS and control of *Mycobacterium avium* (*M. avium*) infection in human primary macrophages. Using chemical inhibition, targeted mass spectrometry and single cell image analysis, we showed that macrophages infected with *M. avium* switch to aerobic glycolysis without any major imbalances in the tricarboxylic acid cycle volume or changes in the energy charge. Instead, we found that pyruvate import contributes to hyperpolarization of mitochondria in infected cells and increases production of mitochondrial reactive oxygen species by the complex I *via* reverse electron transport, which reduces the macrophage burden of *M. avium*. While mycobacterial infections are extremely difficult to treat and notoriously resistant to antibiotics, this work stresses out that compounds specifically inducing mitochondrial reactive oxygen species could present themself as valuable adjunct treatments.

## Introduction

Metabolic reprogramming is a key feature of activated macrophages in which the energy production shifts from oxidative phosphorylation to aerobic glycolysis (1). Our understanding of the role of mitochondria has recently shifted from sole energy production through oxidative phosphorylation to production of specific signaling metabolites contributing to the anti-microbial and inflammation response. As an example, itaconate, which is a product of the decarboxylation of cis-aconitate by the enzyme Immune Response Gene 1 (IRG1), was placed as a central node that controls immune tolerance and trained immunity (2, 3). Further, we and others have independently showed that itaconate, through direct anti-microbial properties, is needed to control viral (4), bacterial (5) and mycobacterial infections (6, 7). In their seminal paper, O’Neill and colleagues have demonstrated that succinate regulates the production of interleukin 1β *via* the transcription factor hypoxia induced factor 1α in mouse macrophages challenged with lipopolysaccharide (LPS) (8). Later, the same group has revealed that mitochondrial reactive oxygen species (mtROS) produced by reverse electron transport (RET) are necessary to initiate and modulate the inflammatory reaction after LPS challenge (9). Using bioenergetic and metabolic flux experiments, Cumming et al. have reported that human primary macrophages harbored a reduced glucose metabolism when infected with *Mycobacterium tuberculosis* (*M. tb*) (10). They did not, however, address if glycolysis has an impact on controlling the intracellular burden. More recently, Hackett et al. have shown that *M. tb* limits glycolytic efficiency by targeting phosphofructokinase activity *via* the induction of the non-coding RNA miR-21. This favors intracellular replication by reducing the production of nitrite species (11). Finally, it was shown that mice and bone marrow-derived macrophage infected with *Mycobacterium avium* (*M. avium*) increase their glucose uptake *via* an interferon-gamma signaling loop (12), yet the role of glycolysis in controlling the infection was not investigated. Therefore, whether and how glycolysis is involved in controlling *M. avium* infection by human primary macrophages remain open questions. Here, we report that macrophages rely on glycolysis and RET to control *M. avium* infection and provide molecular evidence linking pyruvate, the end-product of glycolysis, to anti-mycobacterial mtROS production.

## Results

### Engagement of Glycolysis Is Necessary to Control *M. avium* Infection

**Figure 1:**
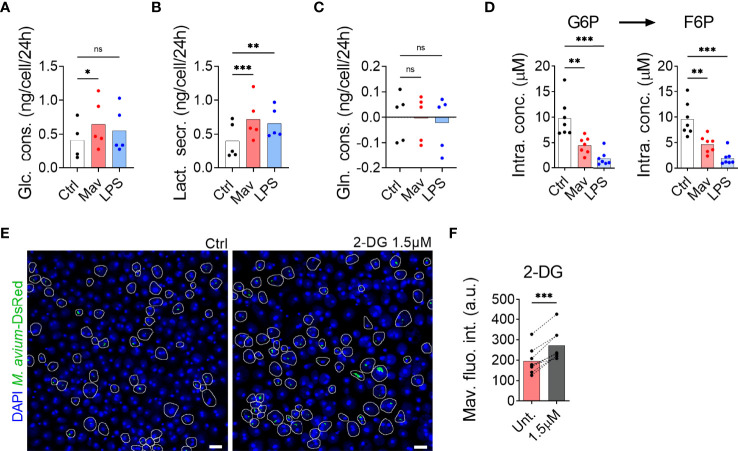
Glycolysis is required to combat *M. avium* infection. Human MDMs were challenged for 24 h with 100 ng/ml LPS (blue bars) or infected with *M. avium*-DsRed (red bars) for 120 min followed by a chase of 24 h **(A–C)**. Glucose (Glc) consumption, lactate (Lac) secretion and glutamine (Gln) consumption were measured using nuclear magnetic resonance. Bar-charts represent average from 5 independent donors. Human MDMs were challenged with 100 ng/ml LPS (blue bars) or infected with *M. avium*-DsRed (red bars) for 10 min followed by a chase of 24 h **(D)**. Intracellular levels (µM) of glucose-6-phosphate (G6P) and fructose-6-phosphate (F6P) were measured in cell extracts using capillary ion chromatography tandem mass spectrometry. Bar-charts represent the average from 7 independent donors. Human MDMs treated with 2-deoxy-glucose (2-DG) were infected with *M. avium*-DsRed for 10 min followed by a chase of 72 h **(E, F)**. Intracellular growth was monitored by confocal microscopy. Dots represent the average fluorescence intensity per individual donor (n > 500 cells per donor and per condition), bar-charts represent the average of 7 independent donors. *P* value between untreated and treated conditions were calculated using the non-parametric ANOVA test **(A–D)** or using the non-parametric paired test Wilcoxon signed-rank test **(F)**. Scale bars represent 10 µm.

To examine whether primary human macrophages switch to glycolysis after infection with *M. avium*, we first measured glucose consumption and lactate secretion in spent medium by nuclear magnetic resonance. **Figure 1** shows a significant increase in glucose uptake (**Figure 1A**) and lactate secretion (**Figure 1B**) without an effect on glutamine uptake (**Figure 1C**), confirming the activation of glycolysis in presence of *M. avium*. The glycolytic switch was further confirmed by targeted mass spectrometric analysis of the intracellular concentration of glucose-6-phosphate (G6P) and fructose-6-phosphate (F6P), the first two intermediates of glycolysis, which revealed a significant decrease in both metabolites when compared to the non-infected control (**Figure 1D**, white and red). Importantly, macrophages treated with 100 ng/ml of LPS for 24 h display the same characteristics of glycolysis engagement (**Figures 1A–D**, blue) as published by others (9), validating our method of measurement. Contrary to Tannahill et al. who reported an increased uptake of glutamine during LPS challenge of mouse macrophages (8), we did not observe an increase of glutamine consumption in LPS-activated human macrophages (**Figure 1C**) which is in accordance with Ko et al. who have reported that human primary macrophages do not rely on glutamine to respond to LPS (13). We next investigated the link between glycolysis and infection by treating infected macrophages with the glycolysis blocker 2-Deoxy-Glucose (2-DG) (**Figure 1E**). Images did not show any signs of cell death induced by the treatment. Quantification of *M. avium* intracellular growth after 72 hours of infection revealed a 40% increase of fluorescence intensity in cells treated with 2-DG, showing that glycolysis is indeed required to control the intracellular burden (**Figure 1F**).

### Mitochondrial Pyruvate Import Is Necessary to Control *M. avium* Infection

**Figure 2:**
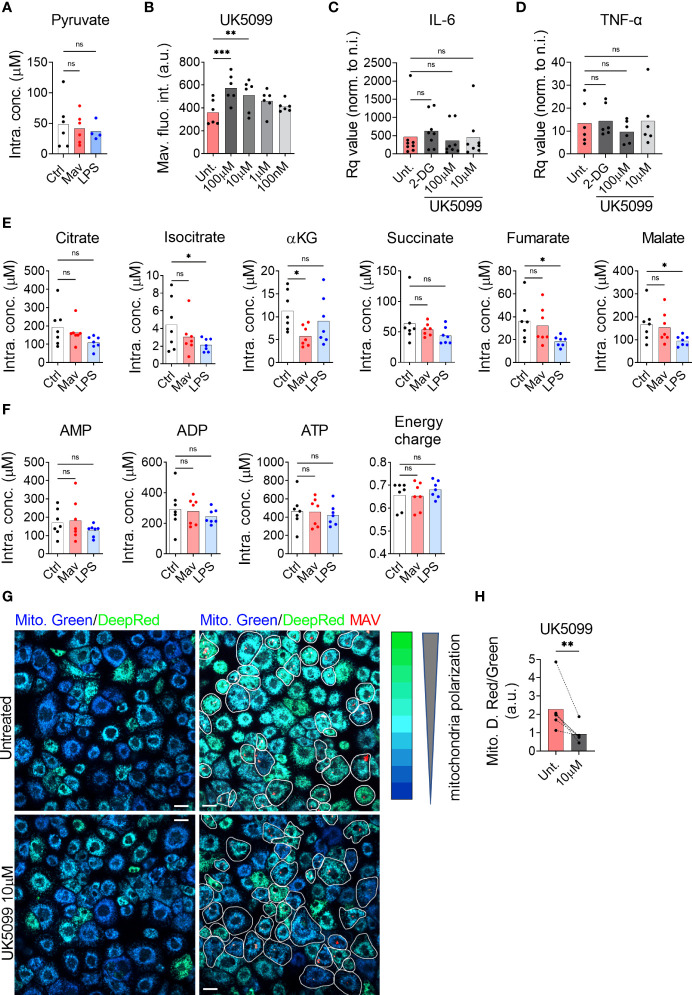
Pyruvate is necessary to maintain mitochondrial hyperpolarization and to control the intracellular burden. Human MDMs were challenged for 24 h with 100 ng/ml LPS (blue bars) or infected with *M. avium*-DsRed (red bars) for 10 min followed by a chase of 24 h **(A)**. Intracellular levels (µM) of pyruvate were measured in cell extracts using liquid chromatography tandem mass spectrometry. Bar-charts represent the average of 6 independent donors. Human MDMs were treated with various concentration of UK5099 and infected with *M. avium*-DsRed for 10 min followed by a chase of 72 h **(B)**. Intracellular growth was monitored by confocal microscopy. Dots represent the average fluorescence intensity per individual donor (n > 500 cells per donor and per condition). Bar-charts represent the average of 6 independent donors. Human MDMs were treated with 1.5 µM 2-DG (dark grey) or 100 or 10 µM UK5099 (grey and light grey) and infected with *M. avium*-DsRed for 10 min followed by a chase of 4 h. Induction of IL-6 **(C)** and TNF-α **(D)** expression were tested by real-time PCR. Bar-charts represent average Rq values from 8 independent donors. Human MDMs were challenged for 24 h with 100 ng/ml LPS (blue bars) or infected with *M. avium*-DsRed (red bars) for 10 min followed by a chase of 24 h **(E, F)**. Intracellular levels (µM) of TCA cycle intermediates **(E)** and adenine nucleotides **(F)** were measured in cell extracts using capillary ion chromatography tandem mass spectrometry. Bar-charts represent average values from 7 independent donors. Human MDMs were treated with 10 µM UK5099 and infected with *M. avium*-DsRed (red) for 10 min followed by a chase of 24 h and mitochondria potential was probed using Mitotracker Green (blue) and DeepRed (green) **(G)**. Merged images are shown. Infected cells are circled in white. Dots represent the average MitoTracker DeepRed/Green fluorescence intensity ratio per individual donor (n > 250 cells per donor and per conditions), bar-charts represent the average of 5 independent donors **(H)**. *P* value between untreated and treated conditions was calculated using the non-parametric ANAOVA test **(A–F)** and the non-parametric paired test Wilcoxon signed-rank test **(H)**. Scale bars represent 10 µm.

The glycolytic pathway leads to the formation of cytosolic pyruvate. Pyruvate can then be converted into lactate by the lactate dehydrogenase in a reaction simultaneously regenerating NAD^+^ from NADH, into alanine by the alanine transaminase, or it can enter the mitochondria *via* the Mitochondrial Pyruvate Carrier (MPC) (14, 15). Quantification by targeted mass spectrometry did not show a significant accumulation in the intracellular level of pyruvate in macrophages infected with *M. avium* or treated with LPS when compared to untreated controls (**Figure 2A**), confirming that pyruvate is metabolized. Mills et al. have demonstrated that during LPS activation, mouse macrophages switch to aerobic glycolysis while repurposing the tricarboxylic acid (TCA) cycle activity to generate specific immunomodulatory metabolites (9), which implies that a fraction of the pyruvate formed by glycolysis enters mitochondria. We therefore hypothesized that pyruvate transport into mitochondria is the first step of the anti-microbial program centered around mitochondrial metabolism. We investigated the potential contribution of mitochondrial pyruvate to the anti-microbial program by treating infected macrophages with the MPC inhibitor UK5099 (16). Quantification of the images revealed a 68% and 46% increase in *M. avium-*DsRed signal in cells treated respectively with 100 µM and 10 µM UK5099 compared to the untreated condition (**Figure 2B**), demonstrating that mitochondrial pyruvate is contributing to control *M. avium* infection in macrophages. We have recently demonstrated that IL-6 and TNF-α, *via* an auto-paracrine loop, help controlling the intracellular burden of *M. avium* by supporting the full activation of the Immune Response Factor 1, which leads to the transcription of the mitochondrial enzyme IRG1 and the local production of the metabolite itaconate (7). To test whether glycolysis inhibition or blockade of pyruvate translocation into mitochondria reduces IL-6 and TNF-α production and therefore increases the intracellular burden, we measured their mRNA level by real-time PCR upon 2-DG and UK5099 treatment. We did not observe any alteration of the induction of IL-6 or TNF-α by the infection when compared to the untreated condition (**Figures 2C, D**), on the contrary, we observed a reduction of IL-1β and IL-10 production upon 2-DG treatment, as shown by Tannahill et al. (**Supplementary Figure 1**) (8). Together, these results demonstrate that pyruvate produced by glycolysis and shuttled in mitochondria contributes directly to the control of the infection.

### Mitochondrial Pyruvate Supports Mitochondrial Hyperpolarization

Once inside the mitochondrial matrix, pyruvate can be converted into acetyl-CoA by the pyruvate dehydrogenase complex (PDC) (17) and feed into the TCA cycle producing reducing equivalents for oxidative phosphorylation. Ruecker et al. have shown that metabolism of fumarate into malate by the mycobacterial enzyme fumarase is required for proper mycobacterial growth but that treatment of *M. tb* culture with additional fumarate was detrimental (18). Further, we and others have previously found that altered intracellular levels of the TCA cycle-derived metabolite itaconate following an infection was indicative of an anti-microbial function (5, 7) Thus, we decided to probe intracellular concentrations of TCA intermediates to search for indications of a potential antimicrobial metabolite. Targeted mass spectrometric quantification showed stable intracellular levels of all measured TCA intermediates after 24 h of infection, except for a significant reduction in the level of alpha-ketoglutarate (αKG) (**Figure 2E**). We next tested if the infection would affect the energy balance of the cells. Targeted mass spectrometric measurement of the adenine nucleotides AMP, ADP, and ATP, did not reveal any changes after 24 h of infection, leaving the adenylate energy charge, reflecting the energy status of a cell (19), unchanged (**Figure 2F**). To test if the infection level was too low to alter the TCA cycle volume and the energy charge, we used an alternative protocol to virtually infect all the macrophages (MOI 10 for 120 min). Using this condition, we did not observe variation in the intracellular metabolite levels (**Supplementary Figure 2**). We therefore concluded that none of the TCA intermediates warranted further investigation to explain the anti-microbial effect of glycolysis. Since the infection did not alter the energy charge nor the TCA cycle volume, we hypothesized that pyruvate import, and mitochondrial activity was geared towards mtROS production *via* the establishment of RET. For this phenomenon to happen, two criteria must be met: the lack of ATP production by oxidative phosphorylation and the maintenance of a high proton motive force/mitochondrial membrane potential (9, 20). To test the engagement of RET, we next treated macrophages infected with *M. avium*-CFP with the potential-insensitive dye MitoTracker Green and the potential-sensitive dye MitoTracker DeepRed (21–23) (**Figure 2G**). Ratiometric measurement showed that the MitoTracker DeepRed signal was significantly increased by *M. avium* infection and reduced by the MPC inhibitor UK5099 (**Figure 2H**), suggesting that pyruvate is involved in the establishment and/or maintenance of high proton motive force induced by the infection.

### Mitochondrial Pyruvate Import Supports RET-Induced mtROS

**Figure 3:**
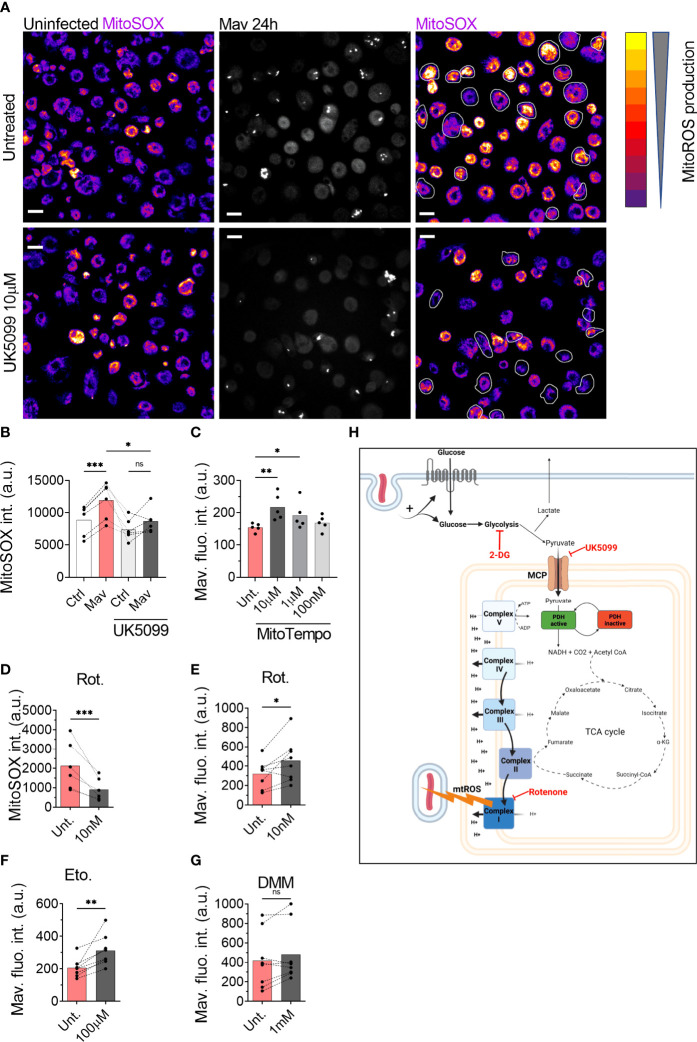
ROS generated by the complex I is necessary to control intracellular burden. Human MDMs were treated with 10 µM UK5099 and infected with *M. avium*-CFP for 10 min followed by a chase of 24 h **(A, B)**. Mitochondrial ROS were stained using MitoSOX Red. Merged images are shown. Dots represent the average MitoSOX Red fluorescence intensity for uninfected (white bars) and infected (red bars) cells (n > 250 cells per donor and per condition). Bar-charts represent the average of 6 independent donors. Infected cells are circled in white. Human MDMs were treated with various concentrations of MitoTEMPO (**C**, red). Intracellular growth was monitored by confocal microscopy. Dots represent the average fluorescence intensity per individual donor (n > 500 cells per donor and per condition), bar-charts represent the average of 5 independent donors. Human MDMs were treated with 10 nM Rotenone (red) and infected with *M. avium*-CFP for 10 min followed by a chase of 24 h **(D)**. Mitochondrial ROS were stained using MitoSOX Red. Dots represent the average MitoSOX Red fluorescence intensity (n > 250 cell per donor and per condition). Bar-charts represent the average of 6 independent donors. Human MDMs were treated with 10 nM Rotenone, 100 µM Etomoxir or 1 mM DMM and infected with *M. avium*-DsRed for 10 min followed by a chase of 24 h (**E–G**, respectively). Intracellular growth was monitored by confocal microscopy. Dots represent the average fluorescence intensity per individual donor (n > 500 cells per donor and per condition), bar-charts represent the average of 7/8 independent donors. *P* value between untreated (grey) and treated conditions (red) was calculated using the non-parametric ANOVA test **(B, C)** or the non-parametric paired test Wilcoxon signed-rank test **(D–G)**. Scale bars represent 10 µm. Working model, generated with Biorender **(H)**.

Given that pyruvate import supports the establishment of a high mitochondrial membrane potential and controls *M. avium* infection, we tested whether pyruvate could be necessary to produce mtROS. Measurement of mtROS production using 500 nM of the mtROS specific dye MitoSOX (24) in the presence of the MPC inhibitor (**Figure 3A**) revealed a significant reduction of the MitoSOX fluorescence intensity in infected macrophages treated with 10 µM UK5099 (**Figure 3B**), indicating that pyruvate shuttling into mitochondria is necessary to produce mtROS. Treatment of infected macrophages with various doses of the mitochondrial specific ROS scavenger MitoTEMPO increased the intracellular burden (**Figure 3C**), confirming that the production of mtROS is an important factor to control the infection. To confirm that mtROS are produced after the engagement of RET, we exploited the ability of the complex I inhibitor Rotenone to reduce the production of mtROS when RET is engaged (25–27). Measurement of mtROS production from macrophages infected with *M. avium*-CFP and co-treated with 10 nM of Rotenone revealed a 50% decrease of the MitoSOX signal over the non-treated condition (**Figure 3D**), demonstrating both the engagement of RET and the role of complex I during the generation of mtROS. To finally demonstrate the role of RET-related mtROS in the control of the intracellular burden, human macrophages infected with *M. avium*-DsRed were treated with 10 nM Rotenone for 72 h. Image quantification revealed a 55% increase of the DsRed signal, indicating a higher intracellular burden (**Figure 3E**). The engagement of complex I was confirmed using a high dose of the Carnitine Palmitoyl Transferase 1 inhibitor Etomoxir as an alternative complex I inhibitor (28, 29). Treatment of infected macrophages with 100 µM Etomoxir increased intracellular burden by 52% after 72 h of infection (**Figure 3F**) whereas treatment with the complex II inhibitor Dimethyl Malonate (9) did not significantly affect the DsRed signal (**Figure 3G**). Further, *M. avium* grown in Middlebrook 7H9 broth in the presence of 2-DG, UK5099, or Rotenone did not show a difference when compared to Middlebrook 7H9 broth alone (**Supplementary Figure 3**). Finally, we verified that the three different compounds do not interfere directly with the DsRed fluorescence intensity and did not observe any significant variation (**Supplementary Figure 4**), demonstrating that the effect of these compounds acts through host factors. Collectively, these data suggest that pyruvate import into mitochondria participates to RET-induced mtROS generated by complex I, which contributes to control the intracellular burden.

## Discussion

In this report, we confirm that glycolysis is important for macrophage defense against mycobacterial infection and describe a central role of pyruvate in linking glycolysis and anti-mycobacterial mtROS production to control the intracellular burden. Alike Cumming et al. who have demonstrated that the non-pathogenic Bacillus Calmette-Guerin and heat-killed *M. tb* increase glycolysis (10), we show that glycolysis is increased in human primary macrophages infected with *M. avium* to facilitate mycobacterial control. We show evidence that the killing mechanism acts through the production of mtROS by the complex I of the electron transport chain *via* the engagement of RET. This mechanism acts in parallel to other immunometabolic defense pathways activated in *M. avium* infected macrophages, such as the production/induction of itaconate *via* the IRF1-IRG1 pathways (7). Importantly, whereas we found the IRF1-IRG1 pathway to be regulated by TNF-α and IL-6, these cytokines did not seem to be involved in regulating the pyruvate-mtROS mechanism described here. Although others have shown that the glycolytic switch in LPS activated macrophages can result in modulation of cytokine responses such as IL-1β, IL-10, our results align with published data showing that glycolysis inhibition does not alter IL-6 and TNF-α production (8) and reinforce that macrophages use several mechanisms to control the infection. We show chemical evidence that mitochondrial import of pyruvate through MPC activity is necessary to generate a high membrane potential and the subsequent production of mtROS. This finding corroborates with a study showing that the treatment of colon cancer cells with interferon-γ increases MPC1/2 expression, pyruvate transport into mitochondria, and finally increases mtROS production (30). Unfortunately, our data do not explain how pyruvate is driving RET and mtROS; if pyruvate targets the electron transport chain directly or is converted (via the TCA cycle) to another metabolite that initiates RET and mtROS. Several hypotheses have been proposed recently to explain the establishment of RET. First, in their recent review, Yin and O’Neill have proposed that complex II is the site of RET establishment in inflammatory macrophages (31). Tannahill et al. have reported an increase of succinate in BMDMs activated by LPS (8) which is oxidized by complex II and engage RET (9). The lack of succinate increases and the absence of effect of the complex II inhibitor DMM on *M. avium* burden suggests that complex II is not involved in driving anti-mycobacterial mtROS in our study. Second, it has been suggested that the arrest of complex V function drives the electrons to flow backward to complex I and therefore establishes RET (32) and that αKG can bind and inhibit complex V in the model organism *Caenorhabditis elegans* (33). αKG was the only TCA metabolite we found significantly changed (decreased) in *M. avium* infected macrophages, however, whether reduced detection of αKG was due to its interaction with complex V, ultimately driving RET and mtROS, remains to be elucidated. Finally, a retrospective study on the effect of Metformin in a cohort of patients infected with *M. tb* found that diabetic patients treated with Metformin were less likely to develop severe symptoms triggered by *M. tb* infection. Using an *in vitro* model, the authors demonstrated that, despite reducing the level of glucose, the capacity of Metformin to engage mtROS production was responsible for part of the protective effect (34). With conventional antibiotic-based regimen against non-tuberculosis mycobacteria infection presenting a success rate ranging between 42% and 60% (35–37), there is an urge to find alternative/combinatory treatments. Our findings suggest a mechanism, summarized in **Figure 3H**, that links glycolysis and mtROS production during *M. avium* infection in human primary macrophages, and brings attention to the possibility of using compounds that specifically engage mtROS production in adjunct host-directed therapies against mycobacterial infections, where lengthy and toxic treatment combined with increasing drug resistance begs for improved therapeutic solutions.

## Material and Methods

### Reagents

The nuclear dye Hoechst 33342 was purchased from Life Technologies. Ultrapure LPS (*E. coli* 0111:B4) was purchased from *In vivo*gen. MitoTEMPO and UK-5099 were purchased from Sigma Aldrich (SML0737; PZ0160). Etomoxir was purchased from Selleckchem (S8244). MitoTracker Green, MitoTracker DeepRed and MitoSOX Red were purchased from Thermo Fisher Scientific (M7514; M22426; M36008).

### Isolation and Differentiation of Human Primary Macrophages

Buffy coats from healthy blood donors were provided by the Blood Bank, St Olav’s Hospital, Trondheim, after obtaining informed consent and with approval by the Regional Committee for Medical and Health Research Ethics (REC Central, Norway, No. 2009/2245). Peripheral blood mononuclear cells (PBMCs) were isolated using density gradient centrifugation (Lymphoprep, Axis-shield PoC). Monocyte-derived macrophages (MDMs) were generated by plastic adherence for 1h in complete RPMI 1640 (680 µM L-Glutamine and 10 mM Hepes, GIBCO) supplemented with 5% pooled human serum (The Blood Bank, St Olavs hospital) at 37°C and 5% CO_2_. After three washing steps with Hank’s Balanced Salt solution (GIBCO), monocytes were cultivated for 6 days with a change of medium at day 3 in complete RPMI 1640/10% human serum and 10 ng/ml recombinant M-CSF (R&D Systems). At day 6 the medium was replaced with complete RPMI 1640/10% human serum and used for experiments on day 7.

### *M. avium* Culture, Macrophage Infection

*M. avium* clone 104 expressing CFP or DsRed (used to quantify intracellular burden by measuring fluorescent intensity) was cultured in liquid Middlebrook 7H9 medium (Difco/Becton Dickinson) supplemented with 0.2% glycerol, 0.05% Tween 80 and 10% albumin dextrose catalase. Cultures were maintained at log phase growth (optical density between 0.3 and 0.6 measured at 600 nm, OD600) in a 180-rpm shaking incubator at 37°C for a maximum of 5 days. At the day of infection, bacteria were washed with PBS, sonicated and passed through a Gauge 25 needle to ensure single-cell suspension before challenging day 7 MDMs for 10 min at a multiplicity of infection of 10 (MOI 10). In experiments for **Supplementary Figure 2**, MDMs were challenged with *M. avium* for 120 min. MDMs were subsequently washed with complete RPMI/10% human serum and maintained in culture for the appropriate time. In some experiments, MDMs were challenged with ultrapure LPS (TLR4; 100 ng/ml) for 24 h.

### Mitochondria and Mitochondrial ROS Live Staining

Human MDMs cultivated on glass-bottomed 96 well plates (IBL) were incubated with 10 nM MitoTracker Green and DeepRed for 10 min at 37°C for mitochondria staining or with 500 nM MitoSOX Red for 10 min at 37°C for mtROS staining. MDMs were then washed with complete RPMI/10% human serum and immediately imaged with a confocal microscope.

### Imaging

MDMs cultivated on glass-bottomed 96 well plates were imaged with a Zeiss LSM880 confocal microscope with 40x NA=1.4 objective (Carl Zeiss Micro-imaging Inc.). Emissions were collected using GaAsP hybride detectors. The following acquisition parameters were used: 1024*1024 pixels image size, numerical zoom set to 0.6, frame averaging 1, and 3D acquisition to collect the entire cell with a Z-stack step of 0.25 µm. CFP was excited with a 458 nm Argon laser and emissions were collected through a 470-500 nm window. MitoTracker Green was excited with a 488 nm Argon laser and emissions were collected through a 505-550 nm window. DsRed and MitoSOX Red were excited with a 543 nm HeNe lasers and emissions were collected through a 560-610 nm window. MitoTracker DeepRed was excited with a 633-diode laser and emissions were collected through a 645-700 nm window. Images were analyzed with Image J (NIH).

### Fluorescence Quantification for *In Situ* CFU, Mitochondrial Potential and Mitochondrial ROS

3D stacks were projected using the “Sum” setting. Resulting images were converted to 8-bit. Regions of interest were drawn around macrophages containing *M. avium*. The background was estimated using HiLo Lookup Tables and subtracted. For mitochondrial potential, MitoTracker DeepRed values were normalized by MitoTracker Green values. A minimum of 250 infected cells per condition and per donor were counted.

### Targeted Mass Spectrometric Absolute Quantification of Intracellular Central Carbon Metabolite Levels

Sampling of MDMs for extraction and mass spectrometric quantification of intracellular central carbon metabolite levels was performed as described for adherent cell lines in (38). In brief, spent medium was discarded and cells were rinsed of extracellular metabolites with cold saline and water on a cold metal block, serving to simultaneously quench metabolism. Next, cells were mechanically detached in cold water:acetonitrile (1:1) and quenched in liquid nitrogen. 5-8 million MDMs were sampled for each donor and treatment. Extraction was performed by cycling between cold water (4°C) and liquid nitrogen, and extracts were concentrated by lyophilization. Absolute quantification of intracellular G6P, F6P, TCA cycle intermediates and adenine nucleotides was performed by capillary ion chromatography tandem mass spectrometry on a Dionex ICS-4000 capillary ion chromatograph (Thermo Scientific) coupled to a Xevo TQ-XS triple quadrupole mass spectrometer (Waters) operating in negative electrospray mode, as described in (39). The modifications described in (40) was applied to allow for isotope dilution and optimized separation of hexose phosphates. Absolute quantification of intracellular pyruvate levels was performed by liquid chromatography tandem mass spectrometry with upfront derivatization, applying an ACQUITY I-Class UPLC coupled to a Xevo TQ-XS triple quadrupole mass spectrometer (Waters) operating in positive electrospray mode, as described in (38). Complete lists of chromatography and mass spectrometry parameters and settings are described in the referenced publications. Data processing was performed in TargetLynx application manager of MassLynx 4.1 (Waters). Absolute quantification was performed by interpolation of calibration curves prepared from serial dilutions of an analytical grade standards calculated by least-squares regression with 1/x weighting. Response factors of all analytical standards and biological extracts were corrected by the corresponding response factor of their U^13^C-labeled isotopologue. Extract concentrations were normalized to seeding density and to the experimental cell volume (3.94 picoliters) measured by a Moxi Z automated cell counter (Orflo) to obtain intracellular concentrations.

### Energy Charge Calculation

Energy charge (EC) was calculated from normalized concentrations of AMP, ADP and ATP using the following formula: EC=(AMP+(0.5*ADP))/(AMP+ADP+ATP) (19).

### Quantification of Extracellular Metabolite Levels

Extracellular glucose, lactate and glutamine levels of human MDMs were quantified by recording 1D proton NMR spectra of concentrated fresh and spent (24 hours) medium and applying the TopSpin module ERETIC2 (Topspin 4.1.1, Bruker) as described in (41). The difference between fresh and spent medium was normalized to seeding density to obtain consumption and secretion per cell per 24 hours.

### RNA Extraction and RT-qPCR Analysis of mRNA Levels

MDMs were washed with cold PBS and lysed in buffer RLT (Qiagen) with 1% β-mercaptoethanol. Total RNA was extracted using RNeasy Mini kit according to the manufacturer’s protocol (Qiagen), including DNase I digestion (RNase-free DNase set, Qiagen). The samples included in the study presented an OD_260/280_ ratio ~ 2 assessed using a ND-1000 spectrophotometer (NanoDrop). cDNA was synthetized from normalized amounts of RNA using the High-Capacity RNA-to-cDNA kit according to manufacturer’s recommendations (Applied Biosystems). qPCR reactions were performed in 20 µl total volume with 10 ng cDNA input, PerfeCta qPCR FastMix, UNG, ROX (Quanta Biosciences) and TaqMan Gene Expression Assays (Applied Biosystems): GAPDH (Hs99999905_m1), TNF-α (Hs00174128_m1), IL-6 (Hs00985639_m1). The targeted genes were amplified with a StepOnePlus Real-Time PCR System and relative quantities of gene expression were calculated using the comparative C_T_ method with GAPDH gene expression as endogenous control.

### Statistical Analysis

Normality was tested for each experiment. Two-tailed *t*-test and analysis of variance (ANOVA) were used on normally distributed data; Mann and Whitney test was used otherwise. Significant *P* values were set as follows: * <0.05, ** <0.01 and *** <0.005. Statistical analyses were performed using GraphPad Prism 8 (GraphPad Software, Inc.).

## Data Availability Statement

The original contributions presented in the study are included in the article/**Supplementary Material**. Further inquiries can be directed to the corresponding authors.

## Author Contributions 

AG conceptualized the project. AG and CL did the cell and infection experiments. AG did the microscopy and image analysis with support of CL. LR did the mass spectrometry and NMR experiments. CL did the gene expression assays. AG, CL, LR, PB, and TF conceived experiments and interpreted results. AG prepared figures. AG wrote the original draft manuscript. All authors contributed to the article and approved the submitted version.

## Funding

This work was supported by grants from the Olav Thon Foundation (90305600/90305601), the Research Council of Norway (231303, 287696, 223255) and a PhD grant from the NTNU Enabling Biotechnology Strategic Program.

## Conflict of Interest

The authors declare that the research was conducted in the absence of any commercial or financial relationships that could be construed as a potential conflict of interest.

## Publisher’s Note

All claims expressed in this article are solely those of the authors and do not necessarily represent those of their affiliated organizations, or those of the publisher, the editors and the reviewers. Any product that may be evaluated in this article, or claim that may be made by its manufacturer, is not guaranteed or endorsed by the publisher.

## Supplementary Material

Supplementary Figure 1 Human MDMs were treated with 1.5 µM 2-DG (dark grey) or 100 or 10 µM UK5099 (grey and light grey) and infected with *M. avium*-DsRed for 10 min followed by a chase of 4 h. Induction of IL-1β and IL-10 expression were tested by real-time PCR. Bar-charts represent average Rq values from 8 independent donors.

Supplementary Figure 2 Human MDMs were challenged with 100 ng/ml LPS (blue bars) or infected with *M. avium*-DsRed (red bars) for 120 min followed by a chase of 24 h. Intracellular levels (µM) of TCA cycle intermediates (**A**) and adenine nucleotides (**B**) were measured in cell extracts using capillary ion chromatography tandem mass spectrometry. Bar-charts represent average values from 7 independent donors.

Supplementary Figure 3
*M. avium* liquid cultures were treated with 1.5 µM 2-DG, 10 µM UK5099 or 10 nM Rotenone. Bars charts show the average optical density measurements (O.D.) at 24 h from 3 independent experiments.

Supplementary Figure 4
*M. avium* liquid cultures were treated with 1.5 µM 2-DG, 10 µM UK5099 or 10 nM Rotenone. Bars charts show the average DsRed intensity normalized to the bacteria size. A minimum of 300 bacteria were analyzed.

## References

[1] Kelly B O’Neill L. Metabolic Reprogramming in Macrophages and Dendritic Cells in Innate Immunity. Cell Res (2015) 25:771–84. doi: 10.1038/cr.2015.68 PMC4493277 26045163

[2] Domínguez-Andrés J Novakovic B Li Y Scicluna BP Gresnigt MS Arts RJW. The Itaconate Pathway Is a Central Regulatory Node Linking Innate Immune Tolerance and Trained Immunity. Cell Metab (2019) 29:211–20. doi: 10.1016/j.cmet.2018.09.003 30293776

[3] O’Neill LAJ Artyomov M. Itaconate: The Poster Child of Metabolic Reprogramming in Macrophage Function. Nat Rev Immunol (2019) 19:273–81. doi: 10.1038/s41577-019-0128-5 30705422

[4] Daniels BP Kofman S Smith JR Norris GT Snyder AG Kolb JP. The Nucleotide Sensor ZBP1 and Kinase RIPK3 Induce the Enzyme IRG1 to Promote an Antiviral Metabolic State in Neurons. Immunity (2019) 50:64–76. doi: 10.1016/j.immuni.2018.11.017 30635240 PMC6342485

[5] Chen M Sun H Boot M Shao L Chang SJ Wang W. Itaconate is an Effector of a Rab GTPase Cell-Autonomous Host Defense Pathway Against Salmonella. Science (2020) 369:450–5. doi: 10.1126/science.aaz1333 PMC8020367 32703879

[6] Nair S Huynh J Lampropoulou V Loginicheva E Esaulova E Gounder AP. Irg1 Expression in Myeloid Cells Prevents Immunopathology During M. Tuberculosis Infection. J Exp Med (2018) 215:1035–45. doi: 10.1084/jem.20180118 PMC5881474 29511063

[7] Gidon A Louet C Røst LM Bruheim P Flo TH. The Tumor Necrosis Factor Alpha and Interleukin 6 Auto-Paracrine Signaling Loop Controls Mycobacterium Avium Infection *via* Induction of IRF1/IRG1 in Human Primary Macrophages. mBio (2021) 12. doi: 10.1128/mBio.02121-21 PMC8546851 34607464

[8] Tannahill GM Curtis A Adamik J Palsson-McDermott EM McGettrick AF Goel G. Succinate is an Inflammatory Signal That Induces IL-1β Through HIF-1α. Nature (2013) 496:238–42. doi: 10.1038/nature11986 PMC4031686 23535595

[9] Mills EL Kelly B Logan A Costa ASH Varma M Bryant CE. Succinate Dehydrogenase Supports Metabolic Repurposing of Mitochondria to Drive Inflammatory Macrophages. Cell (2016) 167:457–70. doi: 10.1016/j.cell.2016.08.064 PMC5863951 27667687

[10] Cumming BM Addicott K Adamson JH Steyn AJC. Mycobacterium Tuberculosis Induces Decelerated Bioenergetic Metabolism in Human Macrophages. eLife (2018). doi: 10.7554/eLife.39169.018 PMC6286123 30444490

[11] Hackett EE Charles-Messance H O’Leary SM Gleeson LE Muñoz-Wolf N Case S. Mycobacterium Tuberculosis Limits Host Glycolysis and IL-1β by Restriction of PFK-M *via* MicroRNA-21. Cell Rep (2020) 30:124–36. doi: 10.1016/j.celrep.2019.12.015 PMC7764301 31914380

[12] Appelberg R Moreira D Barreira-Silva P Borges M Silva L Dinis-Oliveira RJ. The Warburg Effect in Mycobacterial Granulomas is Dependent on the Recruitment and Activation of Macrophages by Interferon-γ. Immunology (2015) 145:498–507. doi: 10.1111/imm.12464 25807843 PMC4515130

[13] Ko JH Olona A Papathanassiu AE Buang N Park KS Costa ASH. BCAT1 Affects Mitochondrial Metabolism Independently of Leucine Transamination in Activated Human Macrophages. J Of Cell Sci (2020) 133:1–12. doi: 10.1242/jcs.247957 PMC7116427 33148611

[14] Bricker DK Taylor E Schell JC Orsak T Boutron A Chen YC. A Mitochondrial Pyruvate Carrier Required for Pyruvate Uptake in Yeast, Drosophila, and Humans. Science (2012) 377:96–100. doi: 10.1126/science.1218099 PMC3690818 22628558

[15] Herzig S Raemy E Montessuit S Veuthey JL Zamboni N Westermann B. Identification and Functional Expression of the Mitochondrial Pyruvate Carrier. Science (2012) 337:93–6. doi: 10.1126/science.1218530 22628554

[16] Hildyard JCW Ammälä C Dukes ID Thomson SA Halestrap AP. Identification and Characterisation of a New Class of Highly Specific and Potent Inhibitors of the Mitochondrial Pyruvate Carrier. Biochemica Biophys Acta (2005) 1707:221–30. doi: 10.1016/j.bbabio.2004.12.005 15863100

[17] Patel MS Nemeria N Furey W Jordan F. The Pyruvate Dehydrogenase Complexes: Structure-Based Function and Regulation. J Biol Chem (2014) 289:16615–23. doi: 10.1074/jbc.R114.563148 PMC4059105 24798336

[18] Ruecker N Jansen R Trujillo C Puckett S Jayachandran P Piroli GG. Fumarase Deficiency Causes Protein and Metabolite Succination and Intoxicates Mycobacterium Tuberculosis. Cell Chem Biol (2017) 24:306–15. doi: 10.1016/j.chembiol.2017.01.005 PMC5357164 28219662

[19] Atkinson DE Walton G. Adenosine Triphosphate Conservation in Metabolic Regulation. Rat Liver Citrate Cleavage Enzyme. J Biol Chem (1967) 242:3239–41. doi: 10.1016/S0021-9258(18)95956-9 6027798

[20] Robb EL PA Eaton S Szibor M Viscomi C James AM Murphy MP. Control of Mitochondrial Superoxide Production by Reverse Electron Transport at Complex I. J Biol Chem (2018) 293:9869–79. doi: 10.1074/jbc.RA118.003647 PMC6016480 29743240

[21] Lugli E Troiano L Ferraresi R Roat E Prada N Nasi M. Characterization of Cells With Different Mitochondrial Membrane Potential During Apoptosis. Cytometry (2005) 68A:28–35. doi: 10.1002/cyto.a.20188 16184612

[22] Zhou R Yazdi A Menu P Tschopp J. A Role for Mitochondria in NLRP3 Inflammasome Activation. Nature (2011) 469:221–5. doi: 10.1038/nature10156 21124315

[23] Greene AW Grenier K Aguileta MA Muise S Farazifard R Haque ME. Mitochondrial Processing Peptidase Regulates PINK1 Processing, Import and Parkin Recruitment. EMBO Rep (2012) 13:378–85. doi: 10.1038/embor.2012.14 PMC3321149 22354088

[24] Dikalov SI Harrison D. Methods for Detection of Mitochondrial and Cellular Reactive Oxygen Species. Antioxid Redox Signaling (2014) 20:372–82. doi: 10.1089/ars.2012.4886 PMC3887411 22978713

[25] Kushnareva Y Murphy A Andreyev A. Complex I-Mediated Reactive Oxygen Species Generation: Modulation by Cytochrome C and NAD(P)+ Oxidation-Reduction State. Biochem J (2002) 368:545–53. doi: 10.1042/bj20021121 PMC1222999 12180906

[26] Hirst J King M Pryde KR. The Production of Reactive Oxygen Species by Complex I. Biochem Soc Trans (2008) 36:976–80. doi: 10.1042/BST0360976 18793173

[27] Zhao RZ Jiang S Zhang L Yu ZB. Mitochondrial Electron Transport Chain, ROS Generation and Uncoupling. Int J Mol Med (2019) 44:3–15. doi: 10.3892/ijmm.2019.4188 31115493 PMC6559295

[28] Divakaruni AS Hsieh W Minarrieta L Duong TN Kim KKO Desousa BR. Etomoxir Inhibits Macrophage Polarization by Disrupting CoA Homeostasis. Cell Metab (2018) 28(3):490–503. doi: 10.1016/j.cmet.2018.06.001 30043752 PMC6125190

[29] Yao C-H Liu G-Y Wang R Moon SH Gross RW Patti GJ. Identifying Off-Target Effects of Etomoxir Reveals That Carnitine Palmitoyltransferase I is Essential for Cancer Cell Proliferation Independent of β-Oxidation. PloS Biol (2018) 16. doi: 10.1371/journal.pbio.2003782 PMC5892939 29596410

[30] Tai YY Cao F Li M Li P Xu T Wang X. Enhanced Mitochondrial Pyruvate Transport Elicits a Robust ROS Production to Sensitize the Antitumor Efficacy of Interferon-γ in Colon Cancer. Redox Biol (2019) 20:451–7. doi: 10.1016/j.redox.2018.10.024 PMC6232645 30439686

[31] Yin M O’Neill L. The Role of the Electron Transport Chain in Immunity. FASEB J (2021) 35:1–13. doi: 10.1096/fj.202101161R 34793601

[32] Scialò F Fernández-Ayala D Sanz A. Role of Mitochondrial Reverse Electron Transport in ROS Signaling: Potential Roles in Health and Disease. Front Physiol (2017). doi: 10.3389/fphys.2017.00428 PMC5486155 28701960

[33] Randall M Chin XF Pai MY Vergnes L Hwang H Deng G. The Metabolite α-Ketoglutarate Extends Lifespan by Inhibiting ATP Synthase and TOR. Nature (2014) 5110:397–401. doi: 10.1038/nature13264 PMC4263271 24828042

[34] Singhal A Jie L Kumar P Hong GS Khee-Shing Leow M Paleja B. Metformin as Adjunct Antituberculosis Therapy. Sci Trans Med (2014) 6:1–10. doi: 10.1126/scitranslmed.3009885 25411472

[35] Xu HB Jiang R Li L. Treatment Outcomes for Mycobacterium Avium Complex: A Systematic Review and Meta-Analysis. Eur J Clin Microbiol Infect Diseases (2014) 33:347–58. doi: 10.1007/s10096-013-1962-1 23979729

[36] Kwak N Park J Kim E Lee C-H Han SK Yim J-J. Treatment Outcomes of Mycobacterium Avium Complex Lung Disease: A Systematic Review and Meta-Analysis. Clin Infect Diseases (2017) 20:1–10. doi: 10.1093/cid/cix517 28582488

[37] Diel R Nienhaus A Ringshausen FC Richter E Welte T Rabe KF. Microbiologic Outcome of Interventions Against Mycobacterium Avium Complex Pulmonary Disease: A Systematic Review. Chest (2018) 153:888–921. doi: 10.1016/j.chest.2018.01.024 29410162

[38] Røst LM Thorfinnsdottir L Kumar K Fuchino K Langørgen IE Bartosova Z. Absolute Quantification of the Central Carbon Metabolome in Eight Commonly Applied Prokaryotic and Eukaryotic Model Systems. Metabolites (2020) 10:1–20. doi: 10.3390/metabo10020074 PMC7073941 32093075

[39] Kvitvang HFN Kristiansen K Bruheim P. Assessment of Capillary Anion Exchange Ion Chromatography Tandem Mass Spectrometry for the Quantitative Profiling of the Phosphometabolome and Organic Acids in Biological Extracts. J Chromatography (2014) 1370:70–9. doi: 10.1016/j.chroma.2014.10.029 25454131

[40] Stafsnes MH Røst L Bruheim P. Improved Phosphometabolome Profiling Applying Isotope Dilution Strategy and Capillary Ion Chromatography-Tandem Mass Spectrometry. J Chromatography (2018) 1083:278–83. doi: 10.1016/j.jchromb.2018.02.004 29571119

[41] Søgaard CK Blindheim A Røst LM Petrović V Nepal A Bachke S. “Two Hits - One Stone”; Increased Efficacy of Cisplatin-Based Therapies by Targeting PCNA’s Role in Both DNA Repair and Cellular Signaling. Oncotarget (2018) 9. doi: 10.18632/oncotarget.25963 PMC6126690 30197755

